# Supplementary material for: The potential of gene drives in malaria vector species to control malaria in African environments
Source: Nat Commun. 2024 Oct 17;15:8976. doi: 10.1038/s41467-024-53065-z (PMC11486997; doi:10.1038/s41467-024-53065-z)
Supplement: Supplementary file 1 — Supplementary Information [file 41467_2024_53065_MOESM1_ESM.pdf]

SUPPLEMENTARY MATERIAL

"The potential of gene drives in malaria vector species to control malaria in African environments"

Penelope A. Hancock*[1], Ace North*[2], Adrian W. Leach[3], Peter Winskill[1], Azra Ghani[1], H. Charles J. Godfray[4], Austin Burt[3], John D. Mumford^[3]

Table of Contents

S1. The role of extinction-recolonisation cycles ... 3

S2. Gene drive frequencies in each square 12 years post-release ... 4

S3. Relationship between malaria transmission and prevalence ... 5

S4. Sources of geospatial data used in models of gene drive dynamics and impacts on malaria ... 6

S5. Variables used in selecting representative environments ... 11

S6. Mosquito metapopulation model ... 12

S6.1 Overview ... 12

S6.2 Modelling the landscape ... 12

6.2.1 Settlement locations ... 12

6.2.2 Settlement localities ... 12

6.2.3 Human population per settlement ... 13

6.2.4 Water courses ... 13

6.2.5 Rainfall ... 13

6.2.6 Species distributions ... 13

S6.3 Local population dynamics ... 13

6.3.1 Juvenile stage ... 13

6.3.2 Setting the area-specific carrying capacities ... 15

6.3.3 Adult stage ... 15

6.3.4 Dispersal ... 15

6.3.5 Gene drive ... 16

6.3.6 Parameters of models of mosquito and gene drive dynamics ... 16

S7. Modelling malaria infection dynamics in humans ... 20

S7.1 Malaria infection dynamics in mosquitoes ... 28

S7.2 Estimating vector abundances ... 29

S7.3 Treatment and Seasonal Malaria Chemoprevention ... 29

S7.4 Insecticide-treated bednets 30
7.4.1 Impacts on vector mortality and blood-feeding 30
7.4.2 Bednet distributions 32
S7.5 Indoor residual spraying 32
S7.6 Vector interactions with insecticidal interventions 34
S7.7 RTS,S vaccines 34
References 36

S1. The role of extinction-recolonisation cycles

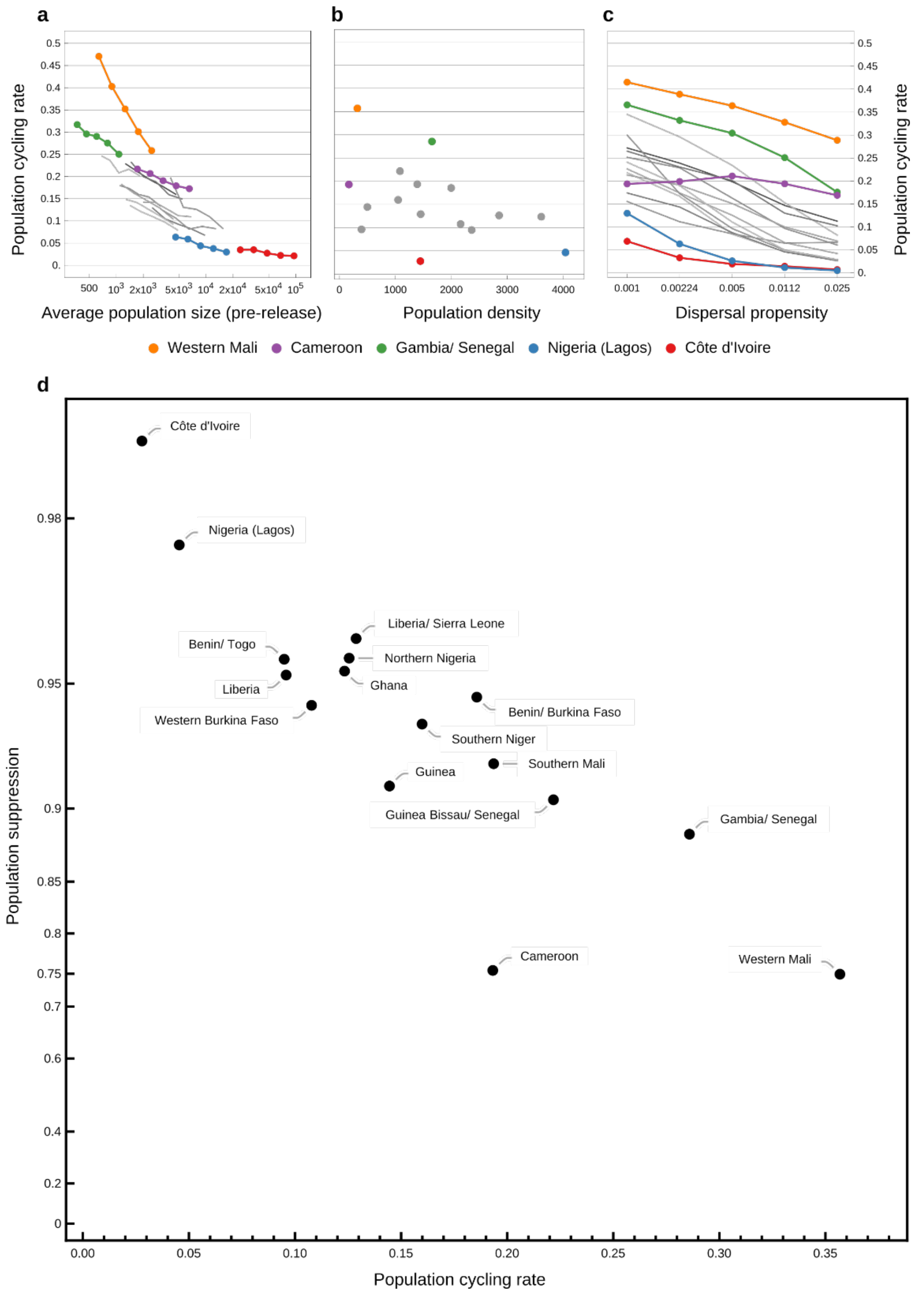

Figure S1. The effects of population size, density, and dispersal, on population cycling. As defined in the main text, population cycling is the rate at which populations cycle through states of extinction and recolonisation. **(a)-(c)**. The rate of population cycling reduces with average population size **(a)**, population density **(b)**, and dispersal propensity **(c)**. **(d)** Population cycling reduces area-wide suppression. All plots use the simulation data for the dominant species from each simulation area (the most abundant species before gene drive releases in each area), from the simulations used to generate Fig. 2 in the main text.

S2. Gene drive frequencies in each square 12 years post-release

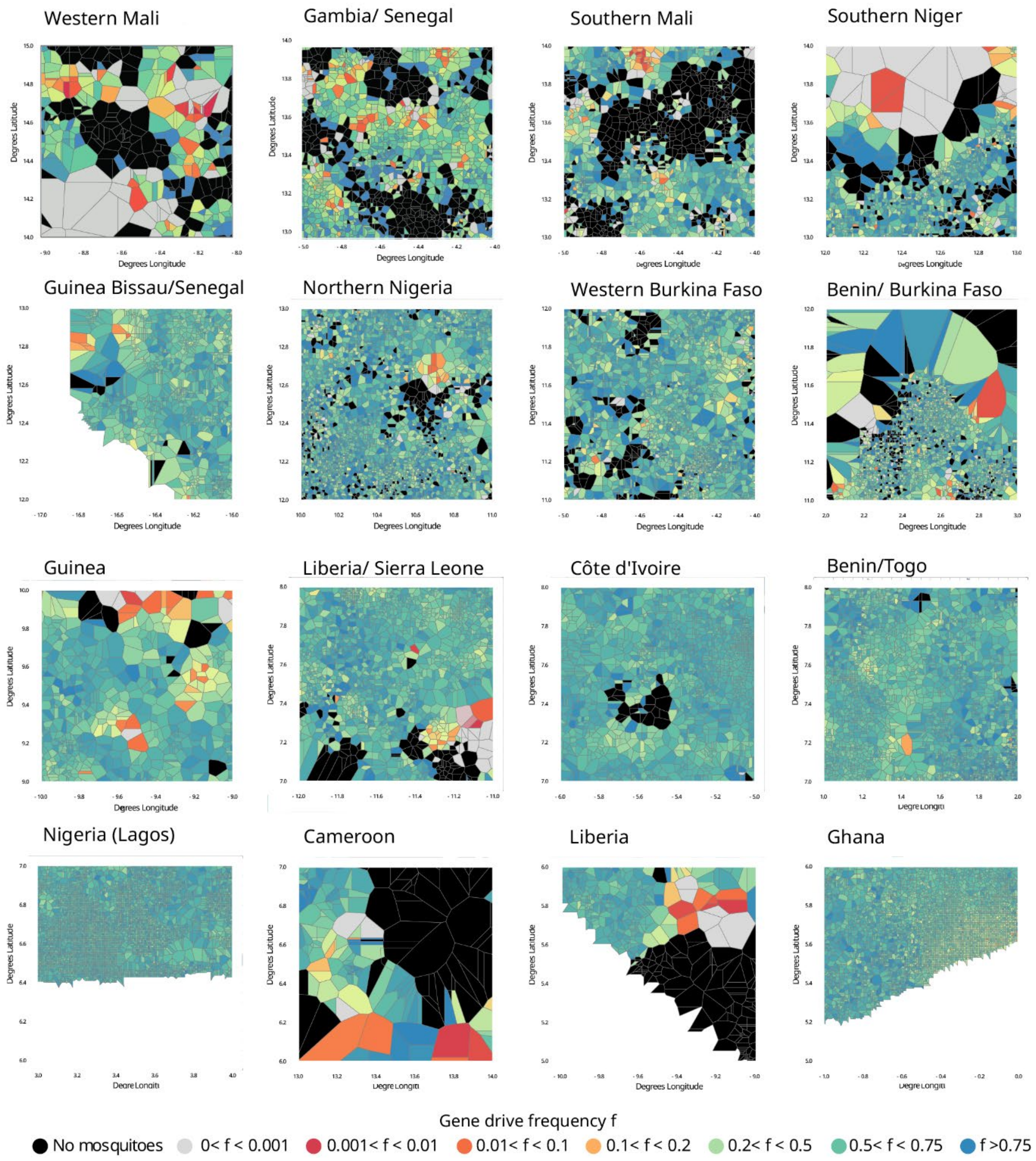

Figure S2. Snapshots of typical simulations of gene drive releases in each of the study areas. In each plot, the localities are coloured by gene drive frequency 12 years after releases in 50 random localities.

S3. Relationship between malaria transmission and prevalence

Malaria transmission rates vary across the modelled areas due to variation in the vector population abundance and species composition, and the coverage of insecticidal vector control interventions (ITNs and IRS) in the human population. The relationship between malaria prevalence in the year immediately prior to the first release of gene drives and the ratio of the total vector population size to the total human population size in each area is non-linear, with a higher gradient occurring at lower values of the number of mosquitoes per human (Figure S2). This pattern is what we would expect to see based on the known relationship between malaria transmission and prevalence [1].

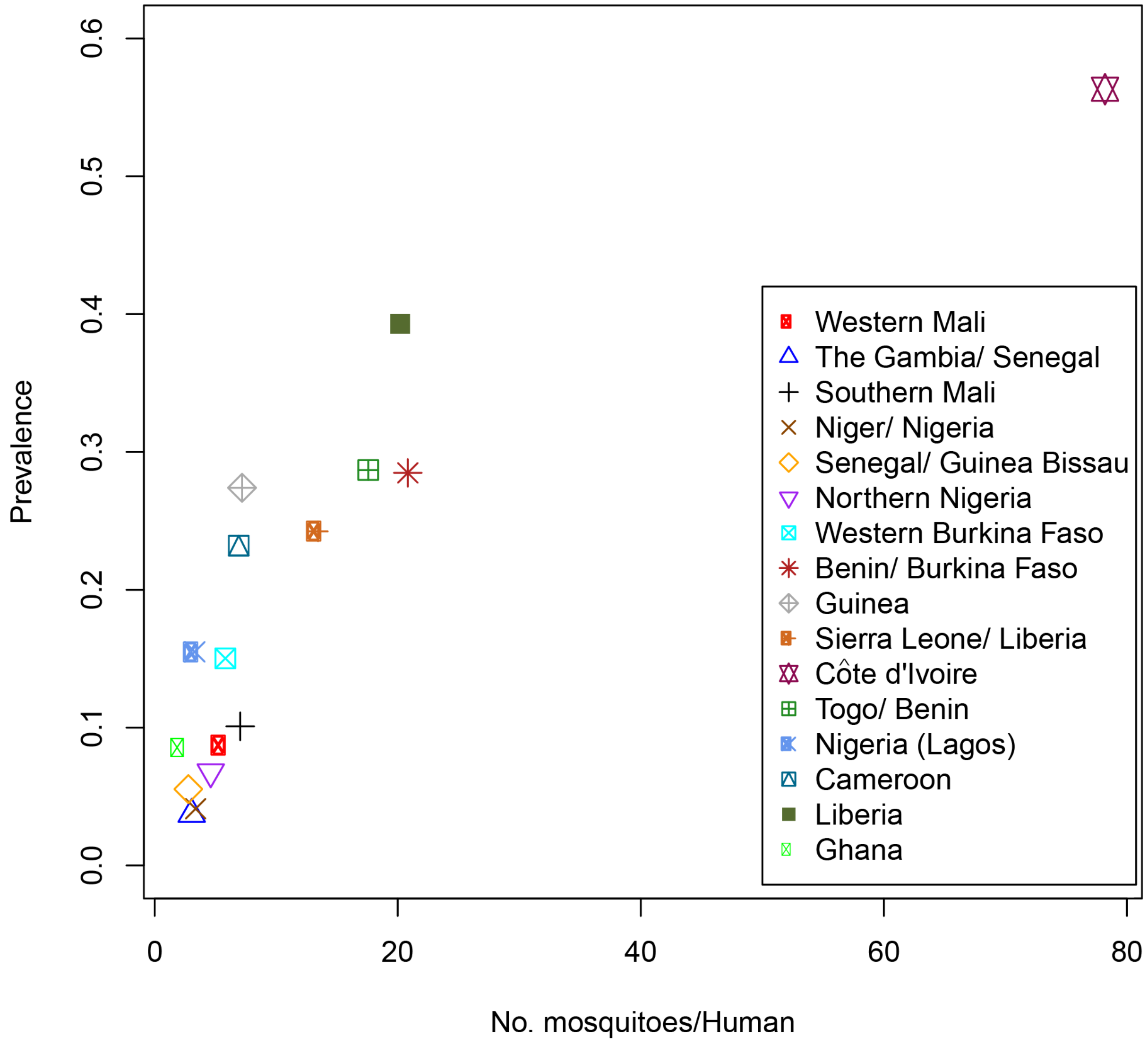

Figure S3. The average annual prevalence of *P. falciparum* malaria predicted by the model vs the average annual total number of vectors per human resident, for each modelled area. Data show predictions from the malaria transmission dynamic model for the year immediately prior to the first releases of gene drives, averaged over *n*=25 simulations.

S4. Sources of geospatial data used in models of gene drive dynamics and impacts on malaria

Sources of geospatial data are provided in Table S1 and the methods used to aggregate fine resolution geospatial data to produce area-level values are described in Table S2.

Table S1. Description of geospatial data sources used in mosquito population and malaria transmission dynamic models

Short name	Description	Temporal resolution	Spatial resolution	Data year	URL	Date accessed	Citation
Plasmodium falciparum prevalence							
Pf prevalence	Proportion of Children 2 to 10 years of age showing, on a given year, detectable *Plasmodium falciparum* parasite	Annual	5km	2019	https://data.malariaatlas.org/maps?layers=Malaria:202206_Global_Pf_Parasite_Rate	01/06/2023	[2]
Vector complex composition							
Arabiensis vs gambiae/coluzzii	Proportional abundance of *An. arabiensis* relative to that of *An. gambiae* and *An. coluzzii* combined	Static	2.5 arcminutes (approx. 5km)	N/A	No URL (sourced from author)	N/A	[3]
Funestus vs An. gambiae complex species	Proportional abundance of *An. funestus* relative to that of *An. gambiae, An. coluzzii* and *An. arabiensis* combined	Static	2.5 arcminutes (approx. 5km)	N/A	No URL (sourced from author)	N/A	[3]
Landscape variables							
Settlement locations	The presence or absence of buildings ('World Settlement Footprint - Sentinel-1/Sentinel-2 - Global, 2019')	Annual	10m	2019	https://download.geoservice.dlr.de/WSF2019/files/	01/06/2023	[4]
Human population	WorldPop human distribution	Annual	1km	2020	https://hub.worldpop.org/geodata/listing?id=64	01/06/2023	[5]

Watercourses	Rivers and lakes	Static	N/A (vector format)	N/A	https://www.hydrosheds.org/hydroatlas	01/06/2023	[6] [7]
Total precipitation	Accumulated water that falls to the Earth's surface (m)	Hourly	0.25 degrees	1979-2021	https://cds.climate.copernicus.eu/cdsapp#!/dataset/reanalysis-era5-single-levels?tab=overview	01/06/2023	[8]
Drug treatment, SMC, vector control							
Treatment seeking	The population at risk weighted mean treatment coverage of an effective antimalarial for each site are taken from the malaria atlas project raster entitled: “Effective treatment with an Antimalarial drug version 2020”	Annual	Admin-1	2000-2018	https://data.malariaatlas.org/maps?layers=Interventions:202106_Global_Antimalarial_Effective_Treatment,Malaria:202206_Global_Pf_Parasite_Rate	01/01/2020	[9-11]
ACT treatment coverage	Proportion of clinical cases that seek treatment that are given ACT	Annual	Admin-1	2000-2018	https://dhsprogram.com/	01/01/2020	[9-11]
SMC coverage	Proportion of 2-5 year olds receiving annual Seasonal Malaria Chemoprevention (SP-AQ)	Annual	Admin-1	2000-2018	https://www.smc-alliance.org/	01/01/2020	[11] SMC Alliance, (2023)
ITN coverage	Proportion of population that sleeps under an ITN during a defined year	Annual	5km	2000-2018	https://data.malariaatlas.org/maps?layers=Interventions:202106_Africa_Insecticide_Treated_Net_Access,Malaria:202206_Global_Pf_Parasite_Rate	01/06/2023	[12]
Coverage of IRS by insecticide class	Coverage of indoor residual spraying with insecticide (proportion of households sprayed)	Annual	Admin-0	2000-2018 Values for the year 2018 were estimated	https://figshare.com/articles/dataset/Indoor_residual_spraying_IRS_coverage_data_from_1997-2017/10066910/1	01/06/2023	[13]

				from the data layer for the year 2017.			
Insecticide resistance of vectors	Phenotypic resistance to insecticides in mosquito species from the An. gambiae complex in Sub-Saharan Africa	Annual	2.5 arcminutes (approx. 5Km)	2005-2017 Values for years 2000-2004 were estimated from the data layer for 2005. Values for the year 2018 were estimated from the data layer for the year 2017.	https://figshare.com/articles/dataset/Mapping_trends_in_insecticide_resistance_phenotypes_in_African_malaria_vectors/9912623	01/06/2023	[14]

Table S2. Methodology for processing geospatial data layers to produce aggregate measures for 1 x 1 degree area

**Short name**	**Aggregation function**	**Aggregate measure per 1 x 1 degree area***	**Use in study**
Rainfall			
Total precipitation	Mean	Average weekly rainfall (mm) within the time period January 1st 1979 to December 31st 2021 at all points on the lattice within our study area (25 points per one- degree grid square).	Area selection
Max dry weeks	Maximum	The maximum of the average number of dry weeks (averaged over years) across all the locations in the square. The threshold for a dry week was less than one mm per week.	Area selection
Plasmodium falciparum prevalence			
Pf prevalence	Mean	Calculated by multiplying together the fine resolution layers for *P. falciparum* prevalence and population size, then summing the quantities across all values (pixels) within each area, and dividing the result by the total population size in each square.	Area selection **and** Parameterising models of gene drive dynamics in mosquitoes and malaria transmission in humans
Vector complex composition			
Proportion *An. arabiensis*	Mean	The average over the area of the abundance of *An. arabiensis* relative to *An. gambiae* and *An. coluzzi* combined	Area selection **and** Parameterising models of gene drive dynamics in mosquitoes and malaria transmission in humans
Proportion *An. funestus*	Mean	The average over the area of the abundance of *An. funestus* relative to *An. arabiensis, An. gambiae* and *An. coluzzi* combined	Area selection **and** Parameterising models of gene drive dynamics in mosquitoes and malaria transmission in humans
Drug treatment, SMC, vector control			

Treatment seeking	Mean weighted by the proportion of each Admin1 unit that intersects each area	The population at risk weighted mean treatment coverage of an effective antimalarial	Parameterising model of malaria transmission in humans
ACT drug treatment coverage	Mean weighted by the proportion of each Admin1 unit that intersects each area	Proportion of clinical cases that seek treatment that are given ACT	Parameterising model of malaria transmission in humans
SMC coverage	Mean weighted by the proportion of each Admin1 unit that intersects each area	Proportion of 2-5 year olds receiving annual Seasonal Malaria Chemoprevention (SP-AQ)	Parameterising model of malaria transmission in humans
ITN coverage	Mean weighted by human population size in each pixel	Proportion of population that sleeps under an ITN during a defined year	Parameterising models of gene drive dynamics in mosquitoes and malaria transmission in humans
Coverage of IRS by insecticide class	Mean weighted by human population size in each pixel	Coverage of indoor residual spraying with insecticide (proportion of households sprayed)	Parameterising models of gene drive dynamics in mosquitoes and malaria transmission in humans
Insecticide resistance of vectors	Mean	Phenotypic resistance to insecticides in mosquito species from the An. gambiae complex in Sub-Saharan Africa	Parameterising models of gene drive dynamics in mosquitoes and malaria transmission in humans

*****Within the set of selected 1° by 1°areas, some areas partially cover the ocean (Figure 1). For these areas, aggregate measures for each variable are calculated using only those pixels that are located on land.

S5. Variables used in selecting representative environments

To capture variation in prevalence across the region, we used a layer of estimates of the *P. falciparum* prevalence in humans in the year 2019 [2]. We calculated the quartiles of the prevalence values across all grid cells in the region. For each area we then calculated the average malaria prevalence in the human population (Table S2) and set a requirement for the selection of areas that four areas must fall within each of the four prevalence quartiles. In addition, our set of selected areas was chosen to capture variation in mosquito population characteristics. Specifically, we aimed to capture variation in mosquito vector species composition, mosquito abundance and seasonality (see the main text). To represent geographic variation in vector species composition we used fine resolution geospatial layers of predictions of the relative abundance of three vector species groups[3], which we used to estimate species composition for each selected area (Table S2). We used two variables describing rainfall patterns to represent geographic variation in the availability and seasonality of larval habitat. The first variable was long term average annual rainfall across each area[8], and the second variable was the spatial maximum of the average number of dry weeks in a year, over all years in the period 1990-2018 (Table S2). To represent variation in human population size across areas we used data from WorldPop on the number of humans residing in each area[5], using data from the year 2020.

We used hierarchical cluster analysis to group all the 1° by 1° areas within our west African region into a set of four clusters with respect to the above five variables describing mosquito population characteristics. Clusters were identified using hierarchical cluster analysis, implemented using the hclust function from R cluster package, with clustering defined by the average Euclidean distance (using hclust with method= “average”). The selected areas show wide variation in the five variables (Figure S4).

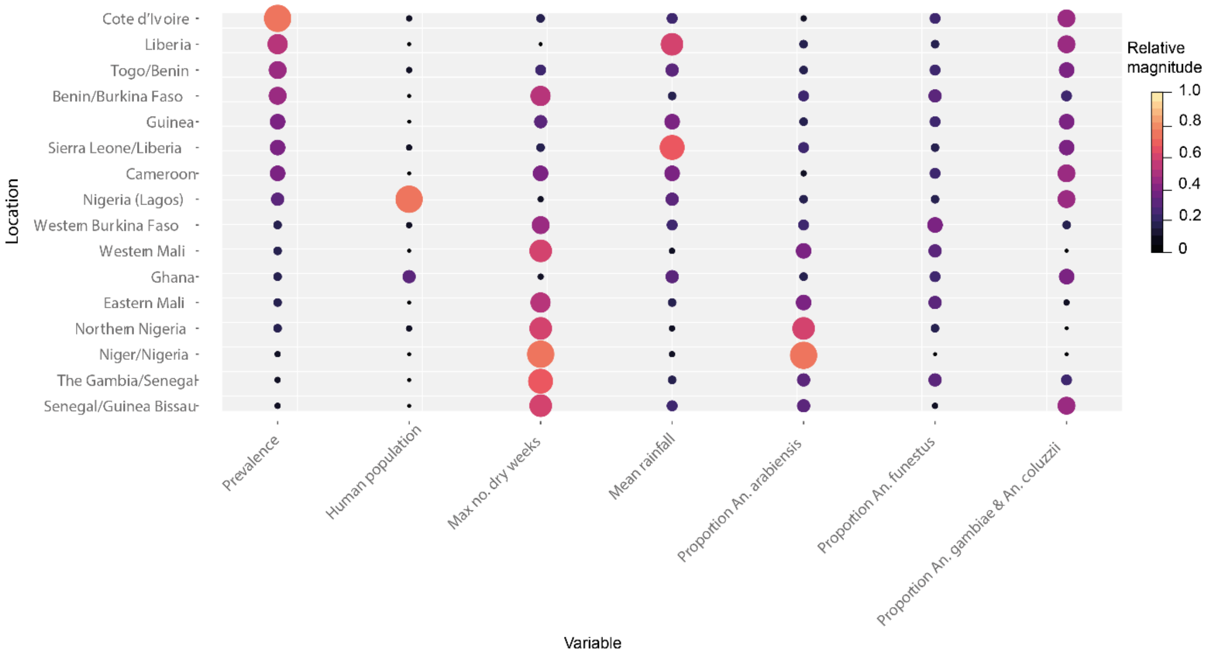

Figure S4. The variation in variables describing malaria prevalence and mosquito populations across the sixteen selected 1° square areas. Colours and sizes of the markers indicate the magnitude of the variable’s value relative to the maximum value of the variable across the sixteen areas.

S6. Mosquito metapopulation model

S6.1 Overview

In this section we fully describe the assumptions of the mosquito metapopulation model. As stated in the main text, the mosquito population is represented as a metapopulation of sub-populations located at the sites of human settlements, for each species and in each area. Each sub-population is composed of juveniles, adult males, virgin females, and mated females. We assume that larval competition affects larval mortality, and increases with larval density to an extent that depends on the amount of larval habitat in a given sub-population. We assume both adult males and mated females may move between neighbouring populations (*dispersal*).

The first part of this description (section 6.2; *Modelling the landscape*) sets out the assumptions relating to landscape. We first describe how the distribution of settlements in each area (henceforth the *landscape*) is inferred from *World Settlement Footprint* data. We next describe how larval habitat is estimated from data representing rainfall, water courses, the human population spatial distribution and, finally, the relative abundances of each species.

The second part (section 6.3; *Local population dynamics*) describes the dynamics of the mosquito sub-populations and how they are affected by larval habitat. We next describe the assumptions of dispersal between sub-populations. Finally, we describe the assumptions that enable us to consider gene drive dynamics in the model; specifically we set out the assumptions of biased inheritance and fitness effects of a gene drive allele at the doublesex locus.

S6.2 Modelling the landscape

Here we describe the input data layers that we used to infer settlement locations across the study area, and the variables affecting the spatial and temporal distribution of larval habitat.

6.2.1 Settlement locations

We inferred locations of high human density ("settlements") using World Settlement Footprint (WSF) data ([4]; Table S1). This data demarks the presence or absence of buildings at 10m resolution, and is available for any location globally. To compute settlement locations, we determined the number of built-up 10m by 10m cells within each square kilometre and defined a square kilometre to be a settlement if this number exceeded a threshold of 0.5%. (i.e. each square km is classified as a settlement if the number of built-up sub-cells exceeds 49 of the 10,000 sub-cells within the square). Note that, in our model, the smallest possible distance between neighbouring settlements is 1km, which occurs if neighbouring squares are both identified as settlements.

This method of settlement inference differs from our previous approach which used settlement databases [15]. We opted for this approach because the WSF dataset has been constructed with uniform methods across the region [16].

6.2.2 Settlement localities

After determining the settlement locations in a given region, we partitioned the region into "localities" by applying a Voronoi tessellation (Fig. 1B). As described below, the localities are used to compute the size of the resident human population associated to each settlement, the length of water courses associated to each settlement, and the dispersal between neighbouring settlements.

6.2.3 Human population per settlement

Human distribution data were obtained from "WorldPop" ([5]; Tables S1), which estimates the human population size in 1km by 1km cells across the globe; we used data for the year 2020. For each settlement, we determined the WorldPop cells with centres within the perimeter of its locality, and summed the population estimates of these cells to obtain a human population estimate for the settlement. We denote by $h_{x,a}$ the estimated human population size in location (x, a) where x is the settlement and a is the area. The variable $h_{x,a}$ is a component of estimating mosquito carrying capacity in this settlement, as described below (see equation 1 below).

6.2.4 Water courses

Watercourse data were obtained from "HydroATLAS", which charts rivers and lakes globally [6,7] (Table S1). The data classifies river segments by their average flow rate, and we re-classified all river segments with flow between 0.1 and 10 m^3s^{-1} as 'seasonal' and segments with flow greater than 10 m^3s^{-1} as 'permanent', though we acknowledge that this classification is approximate. For each settlement x in area a, we denote by $W_{1,x,a}$ the total length of seasonal river segments passing through its locality, and by $W_{2,x,a}$ the sum of the total length of permanent river segments and the total length of lake edge in the locality (we assume lake edges are always permanent). Like human population sizes, these variables contribute to calculating the mosquito carrying capacity for each settlement (see equation 3 below).

6.2.5 Rainfall

Rainfall data were obtained from "ERA5" (Table S1 and S2), which estimates hourly rainfall at all points on a spatial lattice with vertices 0.25 degrees ($\approx 27.5km$) apart from historical observations [8]. We aggregated the data to obtain average weekly rainfall within the time period January 1st 1979 to December 31st 2021 at all points on the lattice within our study area (West Africa). We assume the rainfall at a location (x, a) and on day d, denoted by $\lambda_{x,a,d}$, is given by the rainfall yearly time-series at the nearest point on the aggregated rainfall data lattice. We assume local carrying capacity increases with rainfall (see equation 3 below).

6.2.6 Species distributions

Mosquito species composition was estimated using a predictive map describing the relative abundance of *An. arabiensis* versus *An. funestus* versus *An. gambiae* and *An. coluzzii* (which are combined) in each ≈ 5 x 5km cell (0.0416 x 0.0416 degrees) across the continent [3]. We mapped this onto the settlement locations, by assigning to each settlement the species abundances of the nearest gridcell of the species distribution layer. We assume the carrying capacity for species q in location (x, a) is proportional to relative abundance, which we denote as $\pi_{x,a,q}$ (see equation 3 below).

S6.3 Local population dynamics

6.3.1 Juvenile stage

We model all juvenile mosquito stages (egg, larvae, pupae) as a single class called "juveniles" and denote by $J_{x,a,q,d}$ the number of juveniles in location (x, a) and of species q on day d. Each day, a juvenile will either die or age by one day until it completes development and becomes an adult.

The time required for each juvenile to develop from egg to adulthood is assigned at random from the range 0 to 19 days with probability DEV_τ of requiring τ days. We use as default the distribution given by

$$DEV_{tau} = \begin{cases} 0 \; if \; \tau < 11 \\ 0.1 \; if \; 11 \le \tau \le 20 \end{cases}$$

(1)

such that all juveniles take between 10 and 19 days to develop. Note that fast-developing juveniles are more likely to survive development, so that the adult population will be skewed towards those that developed relatively quickly as juveniles.

The probability that a juvenile of species q in location (x, a) survives day d is

$$p_{x,a,q,d} = \sqrt[15]{\frac{K_{x,a,q,d}}{K_{x,a,q,d} + J_{x,a,q,d}}}\left(1 - \mu_{JUV}\right),$$

(2)

where μ_{JUV} represents density-independent mortality (assumed to be the same for all species groups), and $K_{x,a,q,d}$ is a variable controlling the strength of mortality due to larval competition for resources. The exponent 1/15 is set so a juvenile which requires 15 days to develop will die from larval competition with probability one half, if the number of juveniles in the population is equal to $K_{x,a,q,d}$.

The variable $K_{x,a,q,d}$ varies by location, species, and day, because it is influenced by location-specific factors (the presence of local water bodies, the species distribution and the human population), as well as by rainfall which varies in both time and space. It is computed as:

$$K_{x,a,q,d} = \pi_{x,a,q} DENS^*_{x,a}\left[K_{0,a} + K_{1,a}\left(1 - e^{-\nu_1 \lambda_{x,a,d}}\right) + K_{2,a}\left(1 - e^{-\nu_2\left[W_{1,x,a}\left(1-e^{-\nu_3 \lambda_{x,a,d}}\right)+W_{2,x,a}\right]}\right)\right]$$

(3)

where $DENS^*_x$ relates to the human population density. We set

$$DENS^*_{x,a} = \begin{cases} DENS_{x,a} = \dfrac{h_{x,a}}{AREA_{x,a}} \text{ if } DENS_{x,a} \le N_H \\ N_H \text{ if } DENS_{x,a} > N_H \end{cases}$$

(4)

where $h_{x,a}$ and $DENS_{x,a}$ is the human population size and density at location (x, a) whose area is $AREA_{x,a}$, and N_H is a parameter that ensures mosquito population sizes do not increase indefinitely with human population size. This formulation means that mosquito number increases with the number of humans in small settlements (where the human population density $< N_H$), but will plateau in densely populated settlements such as urban environments.

The three terms within the large square bracket in the equation for $K_{x,a,q,d}$ relate to a baseline level of larval habitat ($K_{0,a}$), the influence of rainfall (scaled by $K_{1,a}$), and the influence of water courses (scaled by $K_{2,a}$), as follows.

We set $\mathrm{K}_{0,\mathrm{a}} > 0$ to assume there is a baseline amount of larval habitat that is present year-round. This ensures that populations typically remain extant through the dry season, in the absence of gene-drive interventions. In previous work, we have explored alternative explanations for dry season persistence such as aestivation and long distance dispersal [15].

The second term represents the contribution to breeding habitats from rainfall on day d, $\lambda_{x,a,d}$, through the creation of puddles and other small water bodies in which anophelines are known to breed. In the absence of rainfall this term is zero, but as precipitation rises it increases at a rate determined by the parameter ν_1 to asymptote at a level $\mathrm{K}_{1,\mathrm{a}}$. Recall that rainfall is averaged by week so that, for any location (x, a), this contribution to larval habitat varies on a weekly rather than daily basis.

The final term represents the larval sites associated with rivers and lakes which may be seasonal ($W_{1,x,a}$) or perennial ($W_{2,x,a}$). We assume that seasonal water courses are replenished by rainfall at a rate determined by the parameter ν_3, providing the same density of breeding habitat as permanent sites when rainfall is heavy. Mosquito carrying capacity increases at a rate determined by the parameter ν_2 as the density of both types of water body grows, the influence reaching an asymptote at $K_{2,a}$.

6.3.2 Setting the area-specific carrying capacities

For each species group, the maximum daily number of adult female mosquitoes in each area a throughout the entire simulation period, $\hat{F}_{a,q}$, was determined by a model of malaria transmission dynamics (described below) which fitted $\hat{F}_{a,q}$ to match predictions of average annual malaria prevalence in humans in each year to data. For each area a, the carrying capacity parameters $K_{1,a}$ and $K_{2,a}$ were adjusted such that population size simulated from the mosquito metapopulation model matched $\hat{F}_{a,q}$. The values of $K_{1,a}$ and $K_{2,a}$ were assumed to be equal for each vector species group.

6.3.3 Adult stage

Juveniles that complete development become male or virgin female adults with equal probability. For a given species q and location (x, a), virgin females mate with probability $\frac{M_{x,a,q,d}}{\beta+M_{x,a,q,d}}$ per day, where $M_{x,a,q,d}$ is the number of males (of that species) in the population and β is the adult male population size at which the daily probability of mating is one half. We assume females only mate at most once during their lifetimes. Each mated female lays a Poisson distributed number of eggs per day until she dies, with expectation that depends on her genotype as described below.

Adult males die with a constant probability μ_{MALE} per day which is assumed to be the same for all species groups. Adult females die with probability $\mu_{FEM_{a,q,d}}$ per day, which may vary by area a, species q and day d. For each area and species group, a time series $\{\mu_{FEM_{a,q,d}}\}$ over the simulation period was computed based on the annual coverage of ITNs and IRS across the human population residing in each area (see below).

6.3.4 Dispersal

Adult mosquitoes move between neighbouring settlements in two ways, which we will refer to as 'neighbourhood dispersal' and 'border dispersal'. Neighbourhood dispersal represents mosquito movements to settlements within a neighbourhood of radius L_D, and occurs at rate ρ_n (the probability that an individual adult mosquito disperses on a given day). We did not consider species-specific differences in this parameter, though for all species groups we simulated the model across a wide range of values to account for uncertainty in dispersal propensity (see Results). If an individual at location (x_1, a) does disperse in this way, the probability that it moves to location (x_2, a) is

$$\frac{L_D - DIST_{x_1,x_2,a}}{\sum_k (L_D - DIST_{x_1 x_k,a})},$$

(5)

where $DIST_{x_1x_2,a}$ is the distance between the two settlements and the sum is over all settlements within the L_D radius (not including the focal settlement). This formulation means that individuals move more frequently between nearby than distant sites.

Border dispersal represents additional movements of mosquitoes between settlements whose localities share a border, and occurs at rate ρ_b (as with neighbourhood dispersal, we do not consider species-specific differences in this parameter). The selection of a destination from these dispersal events is based on the length of shared border. If the initial location is x_1, the probability that destination x_2 is selected is

$$LEN_{x_1x_2,a} \Big/ \sum_k LEN_{x_1x_k,a}$$

(6)

where $LEN_{x_1x_2}$ is the length of the locality border shared by focal settlement x_1 and settlement x_2 and the sum is over all settlements sharing a border with the focal settlement. The inclusion of locality border dispersal in our model ensures that no settlement is entirely unconnected.

6.3.5 Gene drive

Following North et al [17], the model considers three types of allele at the doublesex locus: wildtype (W), gene-drive (G), and a non-functional mutant allele (r2 allele; X) that is resistant to homing. There are therefore six possible mosquito genotypes (W/W, W/G, G/G, W/X, X/X, G/X) and, for each species group, the model keeps track of the numbers of mosquitoes with each genotype in each population. The model also tracks the genotype of the mate of mated females to determine the genotype frequencies in her offspring, resulting in 36 potential types of mated adult female. For each species group, homing occurs during meiosis at a rate $\boldsymbol{e}$, leading to gene drive alleles representing $\frac{\mathbf{1+e}}{\mathbf{2}}$ of the gametes produced by heterozygous (W/D) males and females. Of the remaining $\frac{\mathbf{1-e}}{\mathbf{2}}$ fraction of gametes, a fraction $1 - \chi$ are wildtype, and χ are converted into X alleles by NHEJ repair.

Females that are either fully wildtype (W/W) or heterozygous for the resistant allele (W/X) are assumed to have maximal fertility of l (expected number of eggs per day) owing to having at least one functional doublesex gene (we assume this is the same for each species group). The relative fitness of females that are heterozygous for the gene drive (W/G) is ω ($0 \leq \omega \leq 1$) such that their fertility is $\omega \times l$. The reduced fertility of these individuals may be caused by expression of the transgene in somatic cells, or by parental deposition of the cas9 endonuclease in the sperm of egg [18,19]. Females that lack a copy of the wildtype allele (G/G, X/X, and G/X) are assumed to be sterile. We assume there are no other effects of genotype.

6.3.6 Parameters of models of mosquito and gene drive dynamics

The parameters of the mosquito meta-population model representing the spread dynamics of population suppression gene drives are provided in Table S3.

Table S3. Parameters, variables, and mathematical notation used in the mosquito population model

Parameter/variable	Symbol	Estimate	Units	Reference
**General indices**				
Locality	x	n/a	n/a	n/a
Day	d	n/a	n/a	n/a
Year	y	n/a	n/a	n/a
Species	q	n/a	n/a	n/a
Area	a	n/a	n/a	n/a
Larval duration	τ	n/a	n/a	n/a
**Genotypes**				
Wildtype type mosquitoes	W	n/a	n/a	n/a
Gene drive mosquitoes	G	n/a	n/a	n/a
Resistant mosquitoes	X	n/a	n/a	n/a
**Gene drive parameters**				
Homing rate	e	0.95	$generation^{-1}$	[19]
Proportion of non-homed alleles that form non-functional resistant alleles (R2 alleles)	χ	0.5	n/a	[18]
Relative fitness	ω	0-1	n/a	[18]
**Life-history parameters**				
Larval density independent mortality	μ_{JUV}	0.05	day^{-1}	[15]
Probability that juvenile requires τ days to develop	DEV_τ	$\begin{cases} 0\ if\ \tau < 11 \\ 0.1\ if\ 11 \leq \tau \leq 20 \end{cases}$	n/a	[20,21]
Male mosquito mortality	μ_{MALE}	0.125	day^{-1}	[15]
Female mosquito mortality	$\mu_{FEM_{a,q,d}}$	0.125-0.283	day^{-1}	see Methods
Oviposition rate	l	9	day^{-1}	[15]
**Carrying capacity parameters**				

Baseline contribution to larval habitat	$K_{0,a}$	20	n/a	[15]
Maximum contribution from rain *per se*	$K_{1,a}$	90-16400 across areas	n/a	see Methods
Maximum contribution from water bodies	$K_{2,a}$	90-16400 across areas	n/a	see Methods
Increase in K_0/K_1 per mm rain per week (when rainfall low)	ν_1	0.03	$(mm\ rain)^{-1}(week)^{-1}$	[15]
Increase in K_0/K_2 per km standing water (within locality; when water bodies rare)	ν_2	0.8	$(km\ water)^{-1}$	[15]
Increase in length of standing water from non-permanent waterways per km non-permanent waterways (within locality) per mm rain per week (when rainfall low)	ν_3	0.03	$(mm\ rain)^{-1}(week)^{-1}$	[15]
Threshold human density	N_H	100	$(people)(km)^{-2}$	Expert judgement
Number of males when females mate with probability ½ per day	β	100	n/a	[15]
**Dispersal parameters**				
Dispersal neighbourhood radius	L_D	10	km	Expert judgement
Local dispersal probability	ρ_n	0.005 (0.001-0.025)	$(day)^{-1}$	[15]
Border dispersal probability	ρ_b	$\rho_n/100$	$(day)^{-1}$	Expert judgement
**Variables**				
Settlement area	$AREA_{x,a}$	n/a	$(km)^{-2}$	n/a
Human population in settlement	$h_{x,a}$	n/a	$(people)$	n/a

Human density in settlement	$DENS_{x,a}$	n/a	$(people)(km)^{-2}$	n/a
Adjusted human density in settlement	$DENS^{*}_{x,a}$	n/a	n/a	n/a
River segments seasonal	$W_{1,x,a}$	n/a	(km)	n/a
River segments permanent	$W_{2,x,a}$	n/a	(km)	n/a
Rainfall	$\lambda_{x,a,d}$	n/a	$(mm)(week)^{-2}$	n/a
No. female adult mosquitoes	$F_{x,a,q,d}$	n/a	$(mosquitoes)$	n/a
No. male adult mosquitoes	$M_{x,a,q,d}$	n/a	$(mosquitoes)$	n/a
No. juveniles	$J_{x,a,q,d}$	n/a	$(mosquitoes)$	n/a
Larval survival	$p_{x,a,q,d}$	n/a	$(day)^{-1}$	n/a
Fraction of species	$\pi_{x,a,q}$	n/a	n/a	[3]
Distance between two settlements	$DIST_{x_1,x_2,a}$	n/a	km	n/a
Length of border shared between two settlements	$LEN_{x_1,x_2,a}$	n/a	km	n/a
Maximum daily number of adult female mosquitoes in area	$\hat{F}_{a,q}$	n/a	$(mosquitoes)$	n/a

S7. Modelling malaria infection dynamics in humans

Our model of malaria infection dynamics in humans assumes that the human population within each modelled area a is well-mixed, such that spatial variation in individual infection risk is not considered, and closed, such that immigration and emigration of infections to and from the area is not considered. We model malaria infection and immunity dynamics in humans using the 'malariasimulation' model [22], which is an open-source R-package that implements an individual-based model of malaria infections in humans [23-26]. The full mathematical formulation of 'malariasimulation' is published elsewhere [26,27] and here we provide a summary of the key details. The parameter values that we use in 'malariasimulation' to model human infection and immunity are given in Table S4.

Table S4. Baseline parameters used in 'malariasimulation' to model malaria infection and immunity in humans.

Parameter	Symbol	Estimate	Reference
Human infection duration			
Latent period	d_l	12 days	[28]
Patent infection	d_A	200 days	[28,29]
Clinical disease (treated)	d_T	5 days	[30]
Clinical disease (untreated)	d_D	5 days	[31]
Sub-patent infection	d_U	110 days	[26]
Immunity decay rates			
Inverse of decay rate for maternal immunity to clinical disease	d_M	67.6952 days	[23]
Inverse of decay rate for maternal immunity to severe disease	d_{VM}	76.8365 days	[25]
Inverse of decay rate for acquired pre-erythrocytic immunity	d_B	10 years	[24]
Inverse of decay rate for acquired immunity to clinical disease	d_{cA}	30 years	[32]
Inverse of decay rate for acquired immunity to severe disease	d_{vA}	30 years	[32]
Inverse of decay rate for acquired immunity to detectability	d_{ID}	10 years	[24]
Maternal immunity			
New-born clinical immunity relative to mother	I_{CM}	0.774368	[23]
New-born severe immunity relative to mother	I_{VM}	0.195768	[25]
Probability of pre-erythrocytic infection			
Maximum probability due to no immunity	b_0	0.59	[23]
Maximum reduction due to immunity	b_1	0.5	[24]
Scale parameter	I_{B0}	43.9	[23]
Shape parameter	κ_B	2.16	[23]
Probability of clinical infection			

Maximum probability due to no immunity	ϕ_0	0.792	[23]
Maximum reduction due to immunity	ϕ_1	0.00074	[23]
Scale parameter	I_{C0}	18.02366	[23]
Shape parameter	κ_C	2.36949	[23]
Probability of severe infection			
Maximum probability due to no immunity	θ_0	0.074989	[25]
Maximum reduction due to immunity	θ_1	0.000119	[25]
Scale parameter	I_{V0}	1.09629	[25]
Shape parameter	κ_V	2.00048	[25]
Age dependent modifier	f_{V0}	0.141195	[25]
Age dependent modifier	α_V	2493.41	[25]
Age dependent modifier	γ_V	2.91282	[25]
Immunity reducing probability of detection			
Time-scale at which immunity changes with age	f_{D0}	0.007055	[23]
Scale parameter relating age to immunity	g_D	21.9 years	[23]
Shape parameter relating age to immunity	γ_D	4.8183	[23]
Minimum probability of detection	d_{min}	0.160527	[23]
Scale parameter	I_{D0}	1.577533	[23]
Shape parameter	κ_D	0.476614	[23]
Periods over which immunity is not boosted			
Period in which pre-erythrocytic immunity cannot be boosted	u_B	7.2 days	[23]
Period in which clinical immunity cannot be boosted	u_C	6.06 days	[23]
Period in which severe immunity cannot be boosted	u_V	11.4321 days	[25]
Period in which immunity to detectability cannot be boosted	u_D	9.44512 days	[23]
Infectivity towards mosquitoes			
Infectivity of clinically diseased humans	c_D	0.068	[33]
Infectivity of humans with sub-patent infection	c_U	0.0062	[26]
Infectivity of treated humans	c_T	0.021896	[34]
Age and heterogeneity			
Age dependent biting parameter	g_0	8 years	[35,36]
Age dependent biting parameter	ρ	0.85	[35,36]
Variance of the log heterogeneity in biting rates	σ^2	1.67	[37]

Human demographic and behavioural parameters			
The average age of humans		22 years	[38]
Probability of seeking treatment	$f_{T,a}^{y}$	Varies by area *a* and year *y*	Tables S1, S2
Plasmodium infection dynamics in mosquitoes			
Extrinsic incubation period	T_E	10 days	[39]
Daily rate of seeking a blood meal	f	0.33	[40]
Proportion of bloodmeals taken on humans	Q_0	0.92 (*An. gambiae)*<br>0.71 (*An. arabiensis)*<br>0.94 (*An. funestus*)	[41] (within the maximum and minimum values reported)

Susceptible individuals (i) in area *a* in year *y* experience a force of infection $\Lambda_{i,a}^{y}$ which depends on their exposure to bites from infectious mosquitoes as well as their level of pre-erythrocytic immunity. New-born individuals have a level of maternally-acquired immunity that decays over the first six months of life. Following infection, individuals experience a delay of length d_I representing the period from infection to appearance of blood-stage infection, after which they may either develop clinical disease or asymptomatic infection (*A*). Individuals who develop clinical disease be treated with a probability $f_{T,a}^{y}$, moving to infection state *T*, or will not seek treatment (with probability $1 - f_{T,a}^{y}$) moving to infection state *D*. Those in the latter state eventually resolve symptoms and move to the asymptomatic state (A). Those that are treated subsequently experience a period of drug-dependent partial protection from re-infection (modelled as a Weibull survivorship function) before returning to the fully susceptible state (S). From the asymptomatic state, as parasite density gradually reduces due to the immune response, asymptomatic individuals move to the sub-patent infection state (U) after which they clear infection and return to the susceptible state S. Individuals in states D, A and U can be re-infected (super-infection) and will move into infection states D, T or A following the same process as for primary infection. The transition pathways between these states are depicted in Fig. S5.

We use national life-tables to model death from natural causes [38]. Individuals have age-specific rates at which that are removed from the population that match national life-table rates. Deaths associated with malaria are tracked separately [42]. A new-born individual replaces an individual who has died with matched heterogeneity levels for exposure to infection. In this manner the population size in the simulation remains constant.

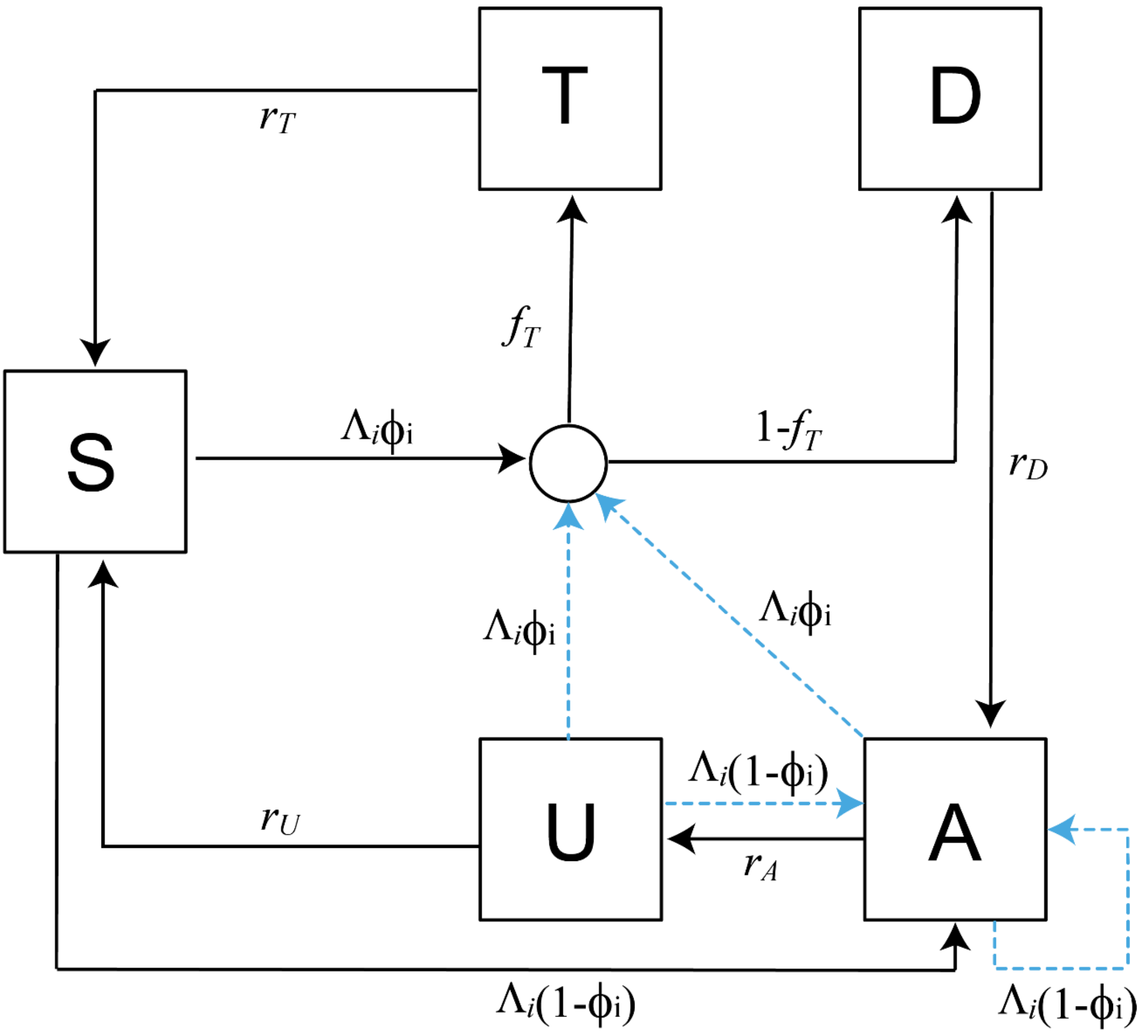

Figure S5. Progression between human infection states. States are shown in boxes and state transitions by arrows with associated hazard rates. The circle represents the treatment node. Superinfection is indicated by the dashed blue arrows. S = susceptible, D = clinical disease, T = successfully treated disease, A = asymptomatic patent infection, U = asymptomatic sub-patent infection. Diagram is reproduced from SI Appendix to Winskill et al. [27].

Heterogeneity in human biting rates

Individual humans experience a unique biting rate which depends on the product of their age-dependent biting rate, $\psi(g)$ and their relative biting rate ζ_i . The relative biting rate for age *g* is defined as:

$$\psi(g) = 1 - \rho\exp\left(-\frac{g}{g_0}\right) \tag{7}$$

where ρ and g_0 are parameters that determine how biting rates increase with age due to increases in body size.

The relative biting rate is drawn from a Log-normal distribution with a mean of 1:

$$\log(\zeta_i) \sim N\left(\frac{-\sigma^2}{2}, \sigma^2\right)$$

(8)

An individual *i* with an age *g* at time *t* then experiences an EIR $\varepsilon_i(g,t)$ of

$$\varepsilon_i(g,t) = \varepsilon_0(g,t)\zeta_i\psi(g)$$

(9)

where $\varepsilon_0(t)$ is the mean EIR experienced by adults at time *t*.

Immunity

Human immunity to infection is modelled as a dynamic process that depends on age and exposure to infection. The maternal immunity that is acquired by infants provides some protection from clinical disease and severe disease at birth is denoted I_{CM} and I_{VM} respectively. These forms of protective maternal immunity decays exponentially at constant rates $r_M = 1/d_M$ and $r_{VM} = 1/d_{VM}$, respectively.

Acquired immunity to infection (pre-erythrocytic immunity) develops at older ages, is boosted by one level following each infected bite provided it is at least u_B days since the last exposure, and decays exponentially in between exposures with rate $r_B = 1\backslash d_B$. Effectively, acquired immunity protects a person from developing patent infection following an infectious bite.

Blood stage immunity is assumed to control parasite density and hence affect the probability of developing severe disease, clinical disease and the detectability of asymptomatic infection. Acquired immunity to each of severe disease, clinical disease and detectability of infection is tracked separately, is boosted by one level following each patent infection provided it is at least u_V, u_C, u_D days respectively since the last exposure, and decays exponentially between exposures with rate $r_{vA} = 1\backslash d_{VA}$, $r_{cA} = 1\backslash d_{cA}$ and $r_{ID} = 1\backslash d_{ID}$ respectively.

All immunity levels are converted to individual time-dependent probabilities using Hill functions. The probability that individual i who is exposed to an infectious bite at time t develops a patent infection is given by

$$b_i(t) = b_0\left(b_1 + \frac{1 - b_1}{1 + \left(\frac{I_B(i,t)}{I_{B_0}}\right)^{\kappa_B}}\right)$$

(10)

where b_0 is the probability of infection with no immunity, $b_0\, b_1$ is the minimum probability, I_{B0} and κ_B are scale and shape parameters respectively and $I_B(i,t)$ is the level of pre-erythrocytic immunity of individual *i* at time *t.*

The probability that an individual *i* who has been infected develops clinical disease at time *t* is

$$\phi(t) = \phi_0\left(\phi_1 + \frac{1 - \phi_1}{1 + \left(\frac{I_{CA}(i,t) + I_{CM}(i,t)}{I_{C0}}\right)^{\kappa_c}}\right)$$

(11)

where ϕ_0 is the probability of disease with no immunity and $\phi_0\phi_1$ is the minimum probability, and I_{c0} and κ_c are scale and shape parameters respectively. Acquired immunity to clinical disease is $I_{CA}(i,t)$ and $I_{CM}(i,t)$ respectively.

The probability that an individual *i* who has been infected develops severe disease at time *t* is

$$\theta_i(g,t) = \theta_0\left(\theta_1 + \frac{1-\theta_1}{1+f_v(i,g)\left(\frac{I_{VA}(i,t)+\ I_{VM}(i,t)}{I_{V0}}\right)^{\kappa_v}}\right)$$

(12)

where θ_0 is the probability of disease with no immunity and $\theta_0\theta_1$ is the minimum probability, and I_{V0} and κ_v are scale and shape parameters respectively. $I_{VA}(i,t)$ is the level of acquired immunity to severe disease and $I_{VM}(i,t)$ is the level of maternal immunity to sever disease of individual *i* at time *t* and

$$f_v(i,g) = 1 - \frac{1-f_{V0}}{1+\frac{\alpha}{\alpha_V}^{\gamma_V}}$$

(13)

is an age-dependent modifier of the risk of severe disease, where f_{V0}, α_V and γ_V are parameters.

Parasite infection can be detected by microscopy. There is a minimum probability of detection d_{min} of an asymptomatic infection in individual *i* of age *g* at time *t* . The capacity to detect infection is determined by:

$$q_i(g,t) = d_1 + \frac{1-d_{min}}{\left(\frac{1+I_D(i,t)}{I_{D0}}\right)^{\kappa_D} f_D(i,g)}$$

(14)

I_{D0} and κ_D are scale and shape parameters respectively, $I_D(i,t)$ is the level of acquired immunity to the detectability of infection of individual *i* at time *t* and

$$f_D(i,g) = 1 - \frac{1-f_{D0}}{1+\left(\frac{g}{g_D}\right)^{\gamma_D}}$$

(15)

represents an age-dependent modifier for infection detectability, where f_{D0}, g_D and γ_D are constant parameters.

Infectivity towards mosquitoes

Individuals in different infection states have a different level of infectiousness towards mosquitoes, with the highest infectivity associated with the states in which parasite density is highest (i.e. disease). Onwards infectiousness is c_A, c_D and c_U in states A, $D$and U respectively, and c_T following treatment.

Stochastic equations

The model is implemented as a stochastic individual-based model using Komolgorov forward equations. The full model equations are provided in [41] following [26] and are reproduced here for completeness. Any individual *i* at time *t* can be placed into a state $\{j, t_T, k, t_k, l, t_l, m, t_m, n, t_n, g, t\}$, where their age g, and infection status *j* (*S, D, A, U, T*), are tracked across time. Here, t_T represents the time of last treatment for individual *i*, *k* depicts the level of infection-blocking immunity and t_k is the time at which infection blocking immunity was last boosted (relating to the last time this individual was exposed to an infectious mosquito bite). The level of the most recent boost of naturally-acquired immunity is denoted l and the time when the exposure occurred t_l . The level of parasite detection immunity is denoted m and the time is t_m . Likewise, *n* and t_n represent the boost and time of exposure for severe disease immunity. The notation I_{mat} and n_{mat} represent these levels and time for maternally-acquired immunity to clinical disease and severe diseases respectively. The Kronecker delta $\delta_{p,q} = 1 \; if \; p = q$ and 0 otherwise.

We let the probability density function be denoted as $P_i(j, t_T, k, t_k, I_{mat}, l, t_l, m, t_m, n_{mat}, n, t_n, g, t)$ for individual i in state $\{j, t_T, k, t_k, I_{mat}, l, t_l, m, t_m, n_{mat}, n, t_n, g, t\}$ at time *t* . Then, the time evolution of the system can be written by the following forward equation:

1
$$\frac{\partial P_i(j, t_T, k, t_k, I_{mat}, l, t_l, m, t_m, n_{mat}, n, t_n, g, t)}{\partial t} + \frac{\partial P_i(j, t_T, k, t_k, I_{mat}, l, t_l, m, t_m, n_{mat}, n, t_n, g, t)}{\partial a} =$$

2
$$\delta_{j,S}[r_t P_i \; + r_U P_i(U, t_T, k, t_k, I_{mat}, l, t_l, m, t_m, n_{mat}, n, t_n, g, t)] +$$

3
$$\delta_{j,A}\left[r_D\left(P_i(D, t_T, k, t_k, I_{mat}, l, t_l, m, t_m, n_{mat}, n, t_n, g, t)\right)\right] +$$

4
$$\delta_{j,U}\left[r_D\left(P_i(A, t_T, k, t_k, I_{mat}, l, t_l, m, t_m, n_{mat}, n, t_n, g, t)\right)\right] +$$

5
$$(1 - b_i)\varepsilon_i(t - d_l)\left[\delta_{j,S} + \delta_{j,D} + \delta_{j,A} + \delta_{j,U}\right]\vartheta_b \circ P_i(j, t_T, k, t_k, I_{mat}, l, t_l, m, t_m, n_{mat}, n, t_n, g, t) +$$

6
$$b_i\varepsilon_i(t - d_l)\left[\delta_{j,A}\left(1 - \phi_i(t)\right) + \delta_{j,D}\phi_i(t)(1 - f_T) + \delta_{j,T}\phi_i(t)f_T\right] \vartheta_I \circ \vartheta_b \circ \vartheta_c \circ \vartheta_d \circ \vartheta_v \circ \sum_{j'} s_{i,j'}(t - t_T)P_i(j', t_T, k, t_k, I_{mat}, l, t_l, m, t_m, n_{mat}, n, t_n, g, t) +$$

7
$$\left[r_B k \frac{\partial}{\partial k} + r_{CA} I \frac{\partial}{\partial I} + r_M I_{mat} \frac{\partial}{\partial I_{mat}} + r_{ID} m \frac{\partial}{\partial m} + r_M n_{mat} \frac{\partial}{\partial n_{mat}} + r_{VA} n \frac{\partial}{\partial n}\right] P_i(j, t_T, k, t_k, I_{mat}, l, t_l, m, t_m, n_{mat}, n, t_n, g, t) +$$

8
$$\mu(g)\delta(g)\delta\left(t_t + T_{big}\right)\delta\left(t_k + T_{big}\right)\delta\left(t_l + T_{big}\right)\delta\left(t_m + T_{big}\right)\delta\left(t_n + T_{big}\right)\delta\left(I_{mat} - mat_c(\zeta_i)\right)\left(n_{mat} - mat_v(\zeta_i)\right) \delta_{i,S}\delta_{k,0}\delta_{l,0}\delta_{m,0}\delta_{n,0}P_i(j, t_T, k, t_k, I_{mat}, l, t_l, m, t_m, n_{mat}, n, t_n, g, t) -$$

9
$$\left[\mu(g) + r_U S_{j,U} + r_D S_{j,D} + r_A S_{j,A} + r_T S_{j,T} + h_i(t - t_E)s_{i,j'}(t - t_T)\right]P_i(j, t_T, k, t_k, I_{mat}, l, t_l, m, t_m, n_{mat}, n, t_n, g, t)$$

(16)

The pathways in Fig. S5 are represented in lines 2-4; the flows into the human compartments (T, U, D and A). The boosting of pre-erythrocytic immunity following exposure is shown in line 5 (for the situation where exposure does not lead to an infection). Line 6 captures the exposure leading to

infection of people in states S, A or U (with onward movement into states A, D or T). The density of individuals in each of the states of immunity are tracked by the communitive integral operators. These operators ϑ_I, ϑ_b , ϑ_c, $\vartheta_d$and ϑ_v are defined below. Line 7 represents the deterministic exponential decay of the respective immunity types. Birth and death processes are indicated in lines 8 and 9.

The commutative integral operators have the following effect on density

$f(j, t_T, k, t_k, I_{mat}, l, t_l, m, t_m, n_{mat}, n, t_n, g, t)$:

$$\vartheta_b \circ f = \delta(t - t_k) \int_0^\infty f(j, t_T, k-1, t_k - u_b - \tau, I_{mat}, l, t_l, m, t_m, n_{mat}, n, t_n, g, t) d\tau + \Phi\left(\frac{t - t_k}{u_b}\right) f(j, t_T, k, t_k, I_{mat}, l, t_l, m, t_m, n_{mat}, n, t_n, g, t)$$

$$\vartheta_c \circ f = \delta(t - t_k) \int_0^\infty f(j, t_T, k, t_k, I_{mat}, l-1, t_l - u_c - \tau, m, t_m, n_{mat}, n, t_n, g, t) d\tau + \Phi\left(\frac{t - t_l}{u_c}\right) f(j, t_T, k, t_k, I_{mat}, l, t_l, m, t_m, n_{mat}, n, t_n, g, t)$$

$$\vartheta_d \circ f = \delta(t - t_k) \int_0^\infty f(j, t_T, k, t_k, I_{mat}, l, t_l, m-1, t_m - u_d - \tau, n_{mat}, n, t_n, g, t) d\tau + \Phi\left(\frac{t - t_d}{u_d}\right) f(j, t_T, k, t_k, I_{mat}, l, t_l, m, t_m, n_{mat}, n, t_n, g, t)$$

$$\vartheta_v \circ f = \delta(t - t_k) \int_0^\infty f(j, t_T, k, t_k, I_{mat}, l, t_l, m, t_m, n_{mat}, n-1, t_n - u_v - \tau, g, t) d\tau + \Phi\left(\frac{t - t_n}{u_v}\right) f(j, t_T, k, t_k, I_{mat}, l, t_l, m, t_m, n_{mat}, n, t_n, g, t)$$

(17)

In these actions, $\Phi(x)$ is an indicator function where

$$\Phi(x) = \begin{matrix} 1 & x < 1 \\ 0 & \text{otherwise} \end{matrix}$$

At birth, *k, l, m* and *n* are set to zero for each individual while t_l, t_T, t_k, t_m and t_n are set to a large negative value $-T_{big}$ (this indicates that there has been no exposure or infection yet). If the previous boost to *k, l, m* and *n* occurred longer than u_b, u_c, u_d and u_v days ago, the naturally-acquired immunity term increase by 1 for each individual receiving an infectious bite (*k*) or who is infected (*l,m* and *n*) respectively. The levels of immunity in individuals aged between 15 and 35 years and within the matched heterogeneity group ζ_i (termed $mat_c(\zeta_i)$ and $t_v(\zeta_i)$) for clinical and severe disease immunity respectively) are used to draw the maternal immunity levels I_{mat} and n_{mat} which are not boosted by exposure. In all cases, immunity levels decay exponentially in the model.

Susceptibility to infection depends on the infection-state and this tracks the prophylactic period of the last drug received. This is defined as:

$$s_{i,j'}(t - t_T) = \begin{matrix} 1 & j' = D, A, U \\ W(t - t_T) & j' = S \\ 0 & j' = T \end{matrix}$$

Here, $W(t - t_T)$ is a Weibull survivorship function modelled to decay over time. The function is specific to the ACT treatment administered.

S7.1 Malaria infection dynamics in mosquitoes

Our model of mosquito population dynamics is a modification of the 'malariasimulation' model (version 1.4.3) such that the rate of emergence of adult mosquitoes is calculated using our spatial metapopulation model and passed to 'malariasimulation' as an external variable, as described in the Methods section of the main text. Here we provide the mathematical formulation of our mosquito population model.

The total number of adult female mosquitoes of species *q* in area *a* at time *t*, $F_a^q(t)$, is subdivided into the numbers that are not infected with the *P. falciparum* parasite, $F_{S,a}^q(t)$, infected with parasites that are still incubating and not yet transmissible, $F_{E,a}^q(t)$, and infectious with transmissible parasites, $F_{I,a}^q(t)$. The dynamics of these three groups of mosquitoes are given by:

$$\frac{dF_{S,a}^q(t)}{dt} = 0.5e_{a,q}(t) - \mu_{FEM_{a,q}}(t)F_{S,a}^q(t) - \Lambda_a^q(t)F_{S,a}^q(t)$$

$$\frac{dF_{E,a}^q(t)}{dt} = \Lambda_a^q(t)F_{S,a}^q(t) - e^{-\mu_{FEM_{a,q}}T_E}F_{E,a}^q(t)(t - T_E) - \mu_{FEM_{a,q}}(t)F_{E,a}^q(t)$$

$$\frac{dF_{I,a}^q(t)}{dt} = e^{-\mu_{FEM_{a,q}}T_E}F_{E,a}^q(t)(t - T_E) - \mu_{FEM_{a,q}}(t)F_{I,a}^q(t)$$

(18)

where $e_{a,q}(t)$ is the daily rate of emergence of adult mosquitoes (see the Methods section in the main text), $\mu_{FEM_{a,q}}(t)$ is the daily rate of adult female mosquito mortality (Table S3), $\Lambda_a^q(t)$ is the daily rate at which species *q* becomes infected with *P. falciparum* in area *a,* and T_E is the entomological inoculation period of the *P. falciparum* parasite (Table S4).

The rate at which adult female mosquitoes become infected is a function of the infectiousness of the human population including an appropriate time-lag d_l to account for the period between humans becoming infected and becoming infectious. The force of infection towards mosquitoes is therefore dependent on all human infected states and is defined as:

$$\Lambda_a^q(t) = \frac{\alpha}{\omega}\iint_0^\infty \zeta\psi(g)(c_D D(\zeta, g, t - d_l) + c_T T(\zeta, g, t - d_l) + c_A A(\zeta, g, t - d_l) + c_U U(\zeta, g, t - d_l))dg d\zeta$$

(19)

where α is the biting rate on humans

$$\alpha = Q_0 f$$

Q_0 quantifies the level of anthropophagy and *f* is the daily rate of taking blood-meals (see Table S4). The parameter ω represents a normalising constant for the biting rate over all ages:

$$\omega = \int_0^\infty \psi(g)G(g)dg$$

(20)

where *G(g)* is the human age distribution.

S7.2 Estimating vector abundances

We estimate e_a, the maximum daily number of adult females that emerge in area *a,* to match model predictions of the annual average prevalence of *P. falciparum* malaria in children aged between 2-10 years (inclusive) for each year in the pre-gene drive period to the yearly data on prevalence [2] (see the main text). Here we give details of the maximum likelihood method used to estimate $\mathrm{e_a}$.
The prevalence for area a in year y obtained from the geospatial layers, $p^y_{MAP,a}$, is calculated as

$$p^y_{MAP,a} = \frac{1}{H^y_a}\sum_{p=1}^{P} p^y_{MAP,p}H^y_{a,p}$$

(21)

where $p^y_{MAP,p}$ is the prevalence in pixel *p* in year *k,* $H^y_{a,p}$ is the number of humans residing in pixel *p* in year *y*, and *P* is the total number of pixels in each area. The area-wide number of humans residing in each area in year *y is* denoted H^y_a.

We fit the model to maximise a modified likelihood function *L* in which more recent observations are more highly weighted compared to earlier observations[43]:

$$L(\{I^y_a\}|\{p^y_{MAP,a}\}) = \prod_{y=0}^{T-1}\left[Bin\left(H^{T-y}_a, p^{T-y}_{MAP,a}\right)\right]^{\gamma^y}, \quad \alpha \in [0,1]$$

(22)

where I^y_a is the number of individuals containing blood stage malaria parasites in the human population residing in area *a* in year *y,* and *T* is the final year of the pre-gene drive period. The parameter γ upweights more recent observations, and was set to a value of γ=0.2. This value of γ was found to produce the minimum out-of-sample root mean square error (RMSE) between the model-predicted malaria prevalences and the data values $p^y_{MAP,a}$ across all areas *a* in the most recent year of the pre-gene drive period, *T.*

S7.3 Treatment and Seasonal Malaria Chemoprevention

As described in the main text, the proportion of clinical cases in each area who receive treatment in a given year, $f^y_{T,a}$, receive either artemether-lumefantrine (AL; a form of artemisinin combination therapy (ACT)) or sulphadoxine-pyrimethamine and amodiaquine (SP-AQ). The probabilities of receiving each treatment type are ACT^k_a and $SPAQ^k_a$ (Tables S1 & S2). In each area and year, a proportion of children aged 2-5 years receive seasonal malaria chemoprevention (SMC), denoted SMC^y_a(Table 1) with the drug SP-AQ. We use the 'set_clinical_treatment' function in 'malariasimulation' to implement the pharmacokinetic-pharmacodynamic (PKPD) models of the time-varying efficacy of each type of drug developed by Okell et al[44], using the parameter values in Table S5.

Table S5. Parameters of the pharmacokinetic-pharmacodynamic model for the time-varying efficacy of artemether–lumefantrine (AL) and sulphadoxine-pyrimethamine and amodiaquine (SP-AQ).

Parameter	Definition	Value	Source
$P_{min,AL}$	Infection probability at infinitely high blood AL concentration	0.95	[44]
$c_{T,AL}$	Infectiousness after AL treatment relative to an untreated infection	0.05094	[44]
w_{AL}	Shape parameter for AL PKPD model	11.3	[44]
λ_{AL}	Scale parameter for AL PKPD model	10.6	[44]
$P_{min,SPAQ}$	Infection probability at infinitely high blood SPAQ concentration	0.9	[44]
$c_{T,SPAQ}$	Infectiousness after SPAQ treatment relative to an untreated infection	0.32	[44]
w_{SPAQ}	Shape parameter for SPAQ PKPD model	4.3	[44]
λ_{SPAQ}	Scale parameter for SPAQ PKPD model	38.1	[44]

S7.4 Insecticide-treated bednets

7.4.1 Impacts on vector mortality and blood-feeding

Our model of the impacts of ITNs on rates of mortality and human blood feeding in the vector population is implemented using the 'set_bednets' function in 'malariasimulation' and is provided on [22]. This model considers that two possible types of ITNs may be used, pyrethroid only and pyrethroid-PBO ITNs. The model parameters relating to ITN impacts are given in Table S6 and a description of how ITN impacts are modelled is below.

Table S6. Model parameters used in the 'set_bednets' function of the 'malariasimulation' model. The levels of pyrethroid resistance in the vector population are estimated using the annual predictive maps developed by Hancock et al. [14].

Parameter	Definition	Value	Source
d_{N0}	Probability of dying during a single feeding attempt	Depends on type of bednet and the level of insecticide resistance, R_a^y. See Table S1.	[41]
r_{N0}	Probability of repeating during a single feeding attempt	Depends on type of bednet and the level of insecticide resistance, R_a^y. See Table S1.	[41]

r_{Nm}	Minimum probability of repeating during a single feeding attempt	0.24	[41]
γ_N	Half-life of insecticidal activity in years	Depends on type of bednet and the level of insecticide resistance, R_a^y. See Table S1.	[41]

The model of the implementation of ITNs used in 'malariasimulation' is described fully in Sherrard-Smith et al. [41] and a summary is provided here. When a person is using a net indoors, we consider that any given Anopheles mosquito feeding attempt can result in either a mosquito being deterred before entering the house or those that enter the house either dying, successful blood-feeding or exiting unfed. These outcomes are summarized as the probabilities of repeating (being deterred or exiting unfed; r_{N0}), dying (d_{N0}) or feeding successfully (1 - $r_{N0} - d_{N0}$). They depend on the level of insecticide resistance in the vector population, the decay in insecticidal activity following net distribution and the type of bednet used. The dependence on the level of pyrethroid resistance, *R*, is incorporated by a non-linear relationship between the probability of surviving exposure to a pyrethroid-only ITN in an experimental hut trial:

$$1 - l_1 = 1/(1 + \frac{R}{\alpha_1})^{-\alpha_2^2}$$

(23)

where l_1 is the probability of dying following ITN exposure (across all mosquitoes that enter a hut), *R* is the probability of dying in a WHO standard pyrethroid susceptibility bioassay and $\alpha_1$and α_2 are constants. The mortality following exposure to a pyrethroid-PBO ITN, l_2, is also dependent on *R* via the following non-linear relationship:

$$l_2 = \frac{1}{1 + \exp\left(-(\beta_1 + \beta_2 l_1\right)}$$

(24)

where $\beta_1$and β_2 are constants. The probabilities r_{N0} and d_{N0} are simple parametric functions of l_1 (for pyrethroid-only nets) and l_2 (for pyrethroid-PBO nets), and other constant parameters including the probability that mosquitoes are not deterred by the presence of a net, *m*, and the probability that mosquitoes enter a house and feed in the absence of an intervention, k_0 [41].

We use the estimates of d_0 and r_0 for each net type across different levels of pyrethroid resistance developed by Sherrard-Smith et al. [41], where resistance is measured by standard susceptibility tests. For each area *a*, we estimate the average level of pyrethroid resistance in the vector population in each year *k*, R_a^y, as:

$$R_a^y = \frac{1}{P}\sum_{p=1}^{P} R_p^y$$

(25)

where R_p^y is the resistance to the pyrethroid deltamethrin in pixel *p* within area *a* in year *y* predicted by the annual geospatial maps of phenotypic resistance to deltamethrin in mosquito species from the *An. gambiae* complex in Sub-Saharan Africa developed by Hancock et al. 2020 [14] (Table S1), and *P* is the total number of pixels in area a.

Insecticidal activity wanes after net distribution. The decay rate of the killing activity of the active ingredient over time, γ_N again depends on the mortalities following exposure to each type of net, l_1 and l_2:

$$\gamma_N = \mu_N + \rho_N(l_1 - \tau)$$

where μ_N and ρ_N are constants that take on different values depending on the type of net, *N* (1 = pyrethroid-only or 2=pyrethroid-PBO) and τ is a constant.

Data to estimate separate values of R_p^y, d_0 and r_0 for different malaria vector species are unavailable, so we use the same values of these parameters for all vector species groups modelled. Different vector species do, however, experience different rates of mortality from ITNs and IRS due to differences between the species in parameters specifying preferences for indoor blood feeding (rather than biting outdoors) and blood feeding on humans (rather than animals) (Tables S4 & S8).

7.4.2 Bednet distributions

The number of new bednets distributed in each area per year is calculated using the 'netz' package in R [45] using the code available on Github [46]. The 'fit_usage' function in 'netz' was used to estimate the annual number of netz distributed to match to the net usage, defined as the proportion of the human population sleeping under a bednet, in each year of the period 2000-2018. Annual net usage for each area was estimated using publish predictive maps [12] (Tables S1 & S2). The model takes into account loss of nets from the human population due to nets being discarded (i.e. thrown away), and assumes a constant rate of net loss per year. We assume that the duration of bednet retention has a half life of 5 years.

S7.5 Indoor residual spraying

Our model of the impacts of IRS on rates of mortality and human blood feeding in the vector population is implemented using the 'set_spraying' function in 'malariasimulation' and is provided on [cite Github]. The parameters that we use in the 'set_spraying' function are given in Table S7. The 'malariasimulation' model of IRS impacts is based on the models developed by Sherrard-Smith et al. [47], and is summarised briefly below.

Table S7. Model parameters used in the 'set_spraying' function of the 'malariasimulation' model. The levels of pyrethroid resistance in the vector population are estimated using the annual predictive maps developed by Hancock et al. [14].

Parameter	Definition	Value	Source
$l_{s\theta}$	The proportion of indoor blood-feeding mosquitos that are killed in a house immediately after spraying	Depends on the insecticide product and the levels of resistance; see Table S1.	[47]
$l_{s\gamma}$	Determines the rate of decay mosquito	Depends on the insecticide product	[47]

	mortality due to IRS in days since spraying	and the levels of resistance; see Table S1.	
$k_{s\theta}$	The proportion of indoor blood-feeding mosquitos successfully blood-feed in a house immediately after spraying	Depends on the insecticide product and the levels of resistance; see Table S1.	[47]
$k_{s\gamma}$	Determines the rate of increase in rates of successful blood feeding with days since spraying	Depends on the insecticide product and the levels of resistance; see Table S1.	[47]
$m_{s\theta}$	The proportion of mosquitoes deterred from entering a sprayed house immediately after spraying	Depends on the insecticide product and the levels of resistance; see Table S1.	[47]
$m_{s\gamma}$	Determines the rate of decay in deterrence with days since spraying	Depends on the insecticide product and the levels of resistance; see Table S1.	[47]

IRS is assumed to impact the proportion of indoor-feeding mosquitoes (Table S8) that are killed (l_s), successfully fed (k_s), or deterred away (m_s) from a sprayed house. These probabilities depend on the insecticide product and the time since spraying. For pyrethroid sprays, they also depend on the level of pyrethroid resistance in the vector population.

Each of these probabilities depends on time since spraying according to the following parametric functions:

$$l_s = \frac{1}{1 + \exp\left(-\left(l_{s\theta} + l_{s\gamma} t\right)\right)}$$

$$k_s = \frac{k_0}{1 + \exp\left(-\left(k_{s\theta} + k_{s\gamma} t\right)\right)}$$

$$m_s = \frac{1}{1 + \exp\left(-\left(m_{s\theta} + m_{s\gamma} t\right)\right)}$$

(26)

where $l_{s\theta}$, $k_{s\theta}$ and $m_{s\theta}$ are values at the time of spraying and $l_{s\gamma}$, $k_{s\gamma}$ and $m_{s\gamma}$ are impact durations.

The association between mosquito mortality measured in WHO standard susceptibility tests and mortality induced by IRS in experimental huts is also used to estimate the diminishing impact of pyrethroid-based IRS with resistance. Sherrard-Smith et al. [47] developed parametric functions similar in form to eqn (23) describing how the values at the time of spraying, $l_{s\theta}$, $k_{s\theta}$ and $m_{s\theta}$, and

the rates of decay $l_{s\gamma}$, $k_{s\gamma}$ and $m_{s\gamma}$ are affected by pyrethroid resistance R_a^k (Table S1). We use the values of pyrethroid resistance in each area for each year, R_a^y, in our parameterisation of pyrethroid IRS effects, assuming that resistance levels are the same for all types of pyrethroid insecticides. We assume that mosquitoes had no resistance to organophosphate or carbamate insecticides. Experimental hut data to parameterise effects of organochlorine IRS insecticides is not available, so we assume that effects of organochlorines are the same as those of pyrethroids. Data describing IRS efficacy for different vector species are unavailable, so we use the same IRS parameterisation for all vector species modelled.

S7.6 Vector interactions with insecticidal interventions

Vector biting behaviour, and how this interacts with the presence of ITNs and IRS, is modelled as described in Griffin et al. [24], who follows the approach taken by Le Menach et al. [48]. This model is parameterised in 'malariasimulation' using the 'set_species' function. The parameter values that we use in 'set_species' are given in Table S8.

Table S8. Vector behaviour parameters used in models of the impacts of ITNs and IRS

Parameter	Description	*An. gambiae* and *An. coluzzii*	*An. arabiensis*	*An. funestus*	Source
Φ_I	Proportion of bites that are taken indoors	0.97	0.96	0.98	[41] (within the maximum and minimum values reported)
Φ_B	Proportion of bites that are taken indoors and bed	0.89	0.9	0.9	[41] (within the maximum and minimum values reported)
k_0	The proportion of mosquitoes that enter a house and successfully feed and exit	0.69	0.69	0.69	[24,41]

S7.7 RTS,S vaccines

We model implementation of RTS,S vaccination based on the recommendation from the World Health Organisation that countries should implement a four-dose schedule in children from five months of age [49]. We use the 'set_rtss_epi' function in 'malariasimulation' to specify that children who reach five months of age are given a three-dose primary series and a booster dose at the same time in the following year. We assume a vaccination coverage of 80%, meaning that 80% of children will receive the primary dose series upon reaching five years of age, and a booster coverage of 64% to represent a drop-off in uptake of boosters. We use the default parameterisation given in 'malariasimulation' of vaccine efficacy, and how this decays over time (Table S9), which is based on the vaccine antibody model developed by Hogan et al.[50].

Table S9. Parameters of the model of RTS,S vaccine antibody model

Parameter	Definition	Value	Source
V_{max}	Maximum efficacy against infection	0.93	[50,51]
α	Dose response shape parameter	0.74	[50,51]
β	Dose response scale parameter	99.4 EU/mL	[50,51]
CSP_{peak}	Peak anti-CSP following the third dose	621 (geometric mean, EU/mL)	[50,51]
CSP_{fourth}	Peak anti-CSP following the fourth dose	277 (mean on log-scale)	[50,51]
ρ_{peak}	Proportion of short-lived response following primary schedule	0.88	[50,51]
ρ_{fourth}	Proportion of short-lived response following the fourth dose	0.7	[50,51]
$1/r_S$	Half-life of short-lived antibodies	45 (mean in days)	[50,51]
$1/r_l$	Half-life of long-lived antibodies	591 (days, sampled from log-normal distribution)	[50,51]

References

1. Beier, J.C., G.F. Killeen, and J.I. Githure, *Short report: Entomologic inoculation rates and Plasmodium falciparum malaria prevalence in Africa.* American Journal of Tropical Medicine and Hygiene, 1999. **61**(1): p. 109-113.
2. Weiss, D.J., T.C.D. Lucas, M. Nguyen, A.K. Nandi, et al., *Mapping the global prevalence, incidence, and mortality of Plasmodium falciparum, 2000-17: a spatial and temporal modelling study.* Lancet, 2019. **394**(10195): p. 322-331.
3. Sinka, M.E., N. Golding, N.C. Massey, A. Wiebe, et al., *Modelling the relative abundance of the primary African vectors of malaria before and after the implementation of indoor, insecticide-based vector control.* Malar J, 2016. **15**.
4. Mattia Marconcini, Annekatrin Metz- Marconcini, Thomas Esch, and N. Gorelick, *Understanding Current Trends in Global Urbanisation - The World Settlement Footprint Suite.* 12th International Symposium on Digital Earth. GI_Forum 2021, 2021. **9**(1): p. 33-38.
5. Lloyd, C.T., H. Chamberlain, D. Kerr, G. Yetman, et al., *Global spatio-temporally harmonised datasets for producing high-resolution gridded population distribution datasets.* Big Earth Data, 2019. **3**(2): p. 108-139.
6. Linke, S., B. Lehner, C. Ouellet Dallaire, J. Ariwi, et al., *Global hydro-environmental sub-basin and river reach characteristics at high spatial resolution.* Sci Dat, 2019. **6**(1): p. 283.
7. Lehner, B., M.L. Messager, M.C. Korver, and S. Linke, *Global hydro-environmental lake characteristics at high spatial resolution.* Sci Dat, 2022. **9**(1): p. 351.
8. Hersbach, H., B. Bell, P. Berrisford, S. Hirahara, et al., *The ERA5 global reanalysis.* Quarterly Journal of the Royal Meteorological Society, 2020. **146**(730): p. 1999-2049.
9. DHS Program. *Demographic and Health Surveys* 2019 [cited 2023; Available from: https://dhsprogram.com.
10. Rathmes, G., S.F. Rumisha, T.C.D. Lucas, K.A. Twohig, et al., *Global estimation of anti-malarial drug effectiveness for the treatment of uncomplicated Plasmodium falciparum malaria 1991–2019.* Malaria J, 2020. **19**(1): p. 374.
11. Winskill, P., *foresite: Access to Malaria Site Files*. 2023 R package version 0.1.0.
12. Bertozzi-Villa, A., C.A. Bever, H. Koenker, D.J. Weiss, et al., *Maps and metrics of insecticide-treated net access, use, and nets-per-capita in Africa from 2000-2020.* Nat Commun, 2021. **12, 3589**(1).
13. Tangena, J.-A., C.J.M. Hendricks, M. Devine, M. Tammaro, et al., *Indoor residual spraying for malaria control in Sub-Saharan Africa 1997 to 2017: an adjusted retrospective analysis.* Malar J, 2019. **19**(150).
14. Hancock, P.A., C.J.M. Hendriks, J.A. Tangena, H. Gibson, et al., *Mapping trends in insecticide resistance phenotypes in African malaria vectors.* PLoS Biology, 2020. **18(6): e3000633**(6).
15. North, A.R. and H.C.J. Godfray, *Modelling the persistence of mosquito vectors of malaria in Burkina Faso.* Malaria Journal, 2018. **17**(1): p. 1-15.
16. Palacios-Lopez, D., F. Bachofer, T. Esch, M. Marconcini, et al., *High-resolution gridded population datasets: Exploring the Capabilities of the world settlement footprint 2019 imperviousness layer for the African continent.* Remote Sensing, 2021. **13**(6): p. 1142.
17. North, A.R., A. Burt, and H.C.J. Godfray, *Modelling the suppression of a malaria vector using a CRISPR-Cas9 gene drive to reduce female fertility.* BMC Biol, 2020. **18**(1).

18. Hammond, A., P. Pollegioni, T. Persampieri, A. North, et al., *Gene-drive suppression of mosquito populations in large cages as a bridge between lab and field.* Nat Commun, 2021. **12**(1): p. 4589.
19. Kyrou, K., A.M. Hammond, R. Galizi, N. Kranjc, et al., *A CRISPR–Cas9 gene drive targeting doublesex causes complete population suppression in caged Anopheles gambiae mosquitoes.* Nat Biotechnol, 2018. **36**(11): p. 1062-1066.
20. Bayoh, M.N. and S.W. Lindsay, *Effect of temperature on the development of the aquatic stages of Anopheles gambiae sensu stricto (Diptera: Culicidae).* Bulletin of entomological research, 2003. **93**(5): p. 375-381.
21. Lyons, C.L., M. Coetzee, and S.L. Chown, *Stable and fluctuating temperature effects on the development rate and survival of two malaria vectors, Anopheles arabiensis and Anopheles funestus.* Parasit Vectors, 2013. **6**(1): p. 1-9.
22. Charles, G., P. Winskill, H. Topazian, J. Challenger, et al. *malariasimulation: An individual-based model for malaria (R package version 1.4.3)*. 2023.
23. Griffin, J.T., N.M. Ferguson, and A.C. Ghani, *Estimates of the changing age-burden of Plasmodium falciparum malaria disease in sub-Saharan Africa.* Nat Commun, 2014. **5**(1): p. 3136.
24. Griffin, J.T., T.D. Hollingsworth, L.C. Okell, T.S. Churcher, et al., *Reducing Plasmodium falciparum Malaria Transmission in Africa: A Model-Based Evaluation of Intervention Strategies.* PLoS Med, 2010. **7**(8).
25. Griffin, J.T., T.D. Hollingsworth, H. Reyburn, C.J. Drakeley, et al., *Gradual acquisition of immunity to severe malaria with increasing exposure.* P Roy Soc B, 2015. **282**(1801).
26. Griffin, J.T., S. Bhatt, M.E. Sinka, P.W. Gething, et al., *Potential for reduction of burden and local elimination of malaria by reducing Plasmodium falciparum malaria transmission: a mathematical modelling study.* Lancet Infect Dis, 2016. **16**(4): p. 465-472.
27. Winskill, P., H.C. Slater, J.T. Griffin, A.C. Ghani, et al., *The US President's Malaria Initiative, Plasmodium falciparum transmission and mortality: A modelling study.* PLoS Med, 2017. **14**(11).
28. Eyles, D.E. and M.D. Young, *The duration of untreated or inadequately treated Plasmodium falciparum infections in the human host.* J Natl Malar Soc, 1951. **10**(4): p. 327-36.
29. Falk, N., N. Maire, W. Sama, S. Owusu-Agyei, et al., *Comparison of PCR-RFLP and Genescan-based genotyping for analyzing infection dynamics of Plasmodium falciparum.* Am J Trop Med Hyg, 2006. **74**(6): p. 944-50.
30. Zwang, J., E.A. Ashley, C. Karema, U. D'Alessandro, et al., *Safety and efficacy of dihydroartemisinin-piperaquine in falciparum malaria: a prospective multi-centre individual patient data analysis.* PLoS One, 2009. **4**(7): p. e6358.
31. Miller, M.J., *Observations on the natural history of malaria in the semi-resistant West African.* Trans R Soc Trop Med Hyg, 1958. **52**(2): p. 152-68.
32. Deloron, P. and C. Chougnet, *Is immunity to malaria really short-lived?* Parasitol Today, 1992. **8**(11): p. 375-8.
33. Collins, W.E. and G.M. Jeffery, *A retrospective examination of mosquito infection on humans infected with Plasmodium falciparum.* Am J Trop Med Hyg, 2003. **68**(3): p. 366-71.
34. Dunyo, S., P. Milligan, T. Edwards, C. Sutherland, et al., *Gametocytaemia after drug treatment of asymptomatic Plasmodium falciparum.* PLoS Clin Trials, 2006. **1**(4): p. e20.
35. Carnevale, P., J.L. Frézil, M.F. Bosseno, F. Le Pont, et al., *[The aggressiveness of Anopheles gambiae A in relation to the age and sex of the human subjects].* Bull World Health Organ, 1978. **56**(1): p. 147-54.
36. Port, G.R., P.F.L. Boreham, and J.H. Bryan, *The relationship of host size to feeding by mosquitoes of the Anopheles gambiae Giles complex (Diptera: Culicidae).* Bull Entomol Res, 1980. **70**(1): p. 133-144.

37. Smith, T., J.D. Charlwood, W. Takken, M. Tanner, et al., *Mapping the densities of malaria vectors within a single village.* Acta Trop, 1995. **59**(1): p. 1-18.
38. United Nations Department of Economic and Social Affairs Population Division (2022), *World Population Prospects 2022: Summary of Results*. 2022: UN DESA/POP/2022/TR/NO. 3.
39. Gu, W., C.M. Mbogo, J.I. Githure, J.L. Regens, et al., *Low recovery rates stabilize malaria endemicity in areas of low transmission in coastal Kenya.* Acta Trop, 2003. **86**(1): p. 71-81.
40. Killeen, G.F., F.E. McKenzie, B.D. Foy, C. Schieffelin, et al., *A simplified model for predicting malaria entomologic inoculation rates based on entomologic and parasitologic parameters relevant to control.* Am J Trop Med Hyg, 2000. **62**(5): p. 535-44.
41. Sherrard-Smith, E., P. Winskill, A. Hamlet, C. Ngufor, et al., *Optimising the deployment of vector control tools against malaria: a data-informed modelling study.* Lancet Planet Health, 2022. **6**(2): p. E100-E109.
42. Walker, P.G., J.T. Griffin, N.M. Ferguson, and A.C. Ghani, *Estimating the most efficient allocation of interventions to achieve reductions in Plasmodium falciparum malaria burden and transmission in Africa: a modelling study.* Lancet Glob Health, 2016. **4**(7): p. e474-84.
43. McCarthy, D. and S.T. Jensen, *Power-weighted densities for time series data.* Ann Appl Stat, 2016. **10**(1): p. 305-334.
44. Okell, L.C., M. Cairns, J.T. Griffin, N.M. Ferguson, et al., *Contrasting benefits of different artemisinin combination therapies as first-line malaria treatments using model-based cost-effectiveness analysis.* Nat Commun, 2014. **5**(1): p. 5606.
45. R Core Team, *R: A Language and Environment for Statistical Computing.* 2022, R Foundation for Statistical Computing, Vienna, Austria. https://www.R-project.org/.
46. Winskill, P., N. Schmidt, and I. Gerard-Ursin, *netz: All Things Bednets*. 2023: R package version 0.3.0.
47. Sherrard-Smith, E., J.T. Griffin, P. Winskill, V. Corbel, et al., *Systematic review of indoor residual spray efficacy and effectiveness against Plasmodium falciparum in Africa.* Nat Commun, 2018. **9(1):4982**.
48. Le Menach, A., S. Takala, F.E. McKenzie, A. Perisse, et al., *An elaborated feeding cycle model for reductions in vectorial capacity of night-biting mosquitoes by insecticide-treated nets.* Malaria J, 2007. **6**(1): p. 10.
49. World Health Organization. *WHO Guidelines for malaria - 14 March 2023*. v6.1 (published on 3/14/23) 2023 01/07/23]; Available from: https://app.magicapp.org/#/guideline/LwRMXj/section/L485Vz.
50. White, M.T., R. Verity, J.T. Griffin, K.P. Asante, et al., *Immunogenicity of the RTS,S/AS01 malaria vaccine and implications for duration of vaccine efficacy: secondary analysis of data from a phase 3 randomised controlled trial.* Lancet Infect Dis, 2015. **15**(12): p. 1450-8.
51. Hogan, A.B., P. Winskill, and A.C. Ghani, *Estimated impact of RTS,S/AS01 malaria vaccine allocation strategies in sub-Saharan Africa: A modelling study.* PLoS Med, 2020. **17**(11).
